# Myocardial tissue characteriation with native myocardial T1 mapping in SLE patients with chest pain

**DOI:** 10.1186/1532-429X-18-S1-W25

**Published:** 2016-01-27

**Authors:** Jaime L Shaw, Mariko L Ishimori, Vaneet Sandhu, Behzad Sharif, Debiao Li, Jay N Schapira, Louise E Thomson, Daniel Wallace, C Noel Bairey Merz, Michael Weisman, Daniel S Berman

**Affiliations:** 1 grid.50956.3f0000000121529905Biomedical Imaging Research Institute, Cedars-Sinai Medical Center, Los Angeles, CA USA; 2 grid.19006.3e0000000096326718Bioengineering, University of California, Los Angeles, Los Angeles, CA USA; 3 grid.50956.3f0000000121529905Department of Medicine, Cedars-Sinai Medical Center, Los Angels, CA USA; 4 grid.50956.3f0000000121529905Barbra Streisand Women's Heart Center, Cedars-Sinai Medical Center, Los Angeles, CA USA

## Background

Systemic Lupus Erythematosus (SLE) patients often exhibit signs and symptoms of cardiac ischemia with an overall increased prevalence of coronary artery disease (CAD), coronary microvascular dysfunction and myocarditis in this population. Potentially, these processes may be associated with subclinical changes in myocardial tissue. Elevated native myocardial T1 and extracellular volume (ECV), measures of myocardial fibrosis, have previously been shown in asymptomatic SLE patients, implying subclinical myocardial disease. We assessed the hypothesis that native myocardial T1 and ECV would be abnormally elevated in SLE subjects with chest pain.

## Methods

We evaluated 13 women with SLE and with current or prior history of chest pain and no obstructive coronary disease and 11 matched normal controls using T1 mapping at 1.5T (Siemens Avanto). Native T1 maps were acquired in a single mid-ventricular slice with a 5(3)3 MOLLI sequence; post-contrast T1 maps were acquired in the same slice 10-15 minutes after contrast injection with a 4(1)3(1)2 MOLLI sequence. Healthy controls had a normal exercise stress test and no history of cardiovascular disease.

## Results

The healthy control group (n = 11) and SLE group (n = 13) were well matched in age (50.5 ± 10.4 vs 46.4 ± 11, p = 0.374) and BMI (25.9 ± 4.5 vs 26.0 ± 7.6, p = 0.357). Eight SLE subjects had current or past corticosteroid use and 7 had current or past use of a cytotoxic agent. Seven SLE subjects had current chest pain while 6 were without. Average SLE duration was 19.5 years. Native myocardial T1 values were increased in the women with SLE with current or past chest pain compared to reference control women (1049.4 ± 34.4 vs 1003.2 ± 19.3 ms, p = 0.005) (Figure). ECV values were elevated compared to published values for normal controls using the same T1 mapping sequences at 1.5T (27.2 ± 3.6 vs 25.4 ± 2.5%). Native T1 values were increased in SLE subjects with current chest pain symptoms compared to those without, however not significantly (1056.3 ± 31.1 vs 1041.5 ± 31.1, p = 0.46). No significant difference in ECV was observed between SLE subjects with and without current chest pain symptoms. No significant relationship was shown between native myocardial T1 or ECV and age, BMI, disease duration, or SLEDAI (SLE Disease Activity Index). However, native myocardial T1 trended toward positive correlation with corticosteroid use (r = 0.507, p = 0.077) as well as the use of cytotoxic agents (r = 0.495, p = 0.086).

## Conclusions

Among women with SLE, chest pain, and no obstructive coronary disease, native myocardial T1 measured by CMR is elevated consistent with diffuse fibrosis compared to controls. Further work will validate the presence of diffuse fibrosis with native T1 and ECV in women with SLE, chest pain, and no obstructive CAD.

**Figure 1:**
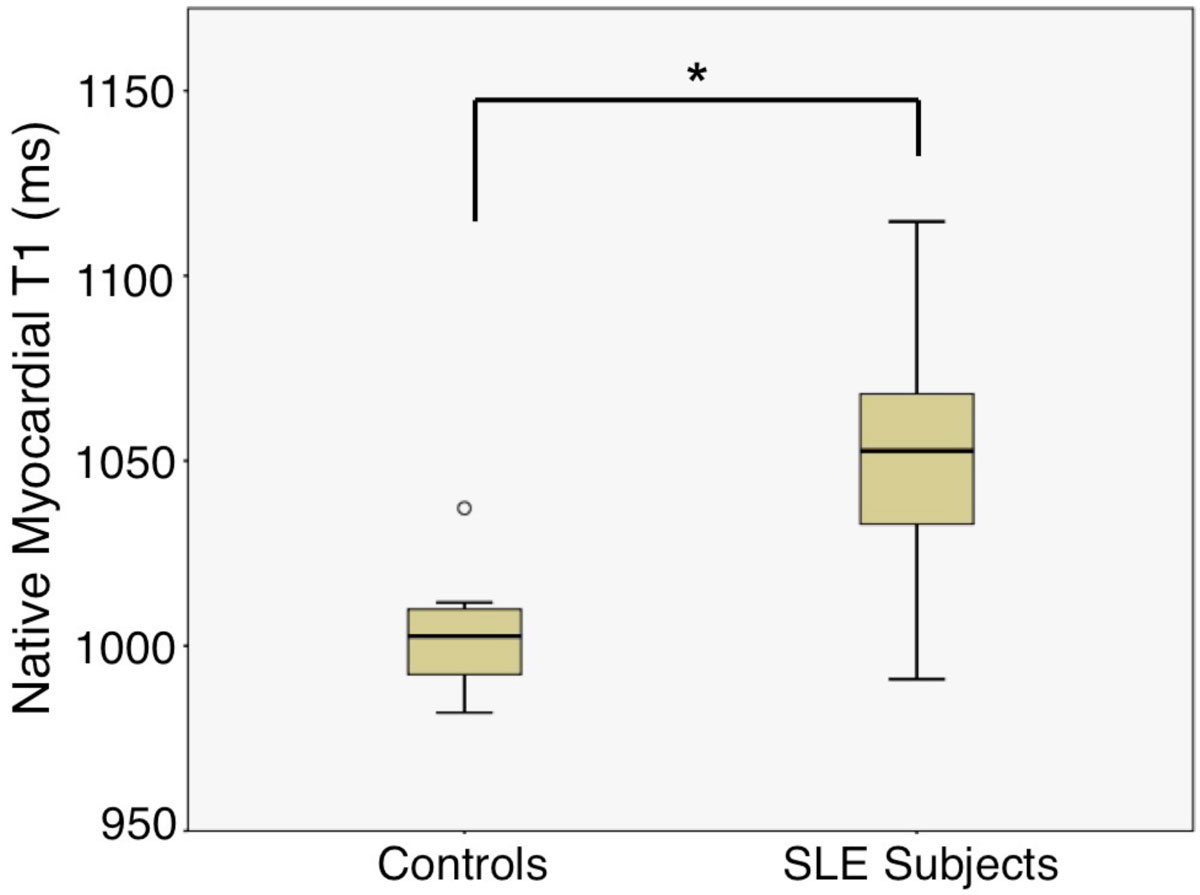
**Native myocardial T1 in SLE subjects with chest pain versus healthy controls (1003.2 ± 19.3 vs 1049.4 ± 34.4 ms, p = 0.005)**.

